# Gender differences in refraction prediction error of five formulas for cataract surgery

**DOI:** 10.1186/s12886-021-01950-2

**Published:** 2021-04-21

**Authors:** Yibing Zhang, Tingyang Li, Aparna Reddy, Nambi Nallasamy

**Affiliations:** 1 Kellogg Eye Center, Department of Ophthalmology and Visual Sciences, University of Michigan, 1000 Wall St, Ann Arbor, MI 48105 USA; 2 Department of Computational Medicine and Bioinformatics, University of Michigan, Ann Arbor, USA

**Keywords:** IOL power calculation, Refraction prediction error, Lens constant optimization

## Abstract

**Objectives:**

To evaluate gender differences in optical biometry measurements and lens power calculations.

**Methods:**

Eight thousand four hundred thirty-one eyes of five thousand five hundred nineteen patients who underwent cataract surgery at University of Michigan’s Kellogg Eye Center were included in this retrospective study. Data including age, gender, optical biometry, postoperative refraction, implanted intraocular lens (IOL) power, and IOL formula refraction predictions were gathered and/or calculated utilizing the Sight Outcomes Research Collaborative (SOURCE) database and analyzed.

**Results:**

There was a statistical difference between every optical biometry measure between genders. Despite lens constant optimization, mean signed prediction errors (SPEs) of modern IOL formulas differed significantly between genders, with predictions skewed more hyperopic for males and myopic for females for all 5 of the modern IOL formulas tested. Optimization of lens constants by gender significantly decreased prediction error for 2 of the 5 modern IOL formulas tested.

**Conclusions:**

Gender was found to be an independent predictor of refraction prediction error for all 5 formulas studied. Optimization of lens constants by gender can decrease refraction prediction error for certain modern IOL formulas.

## Background

Cataract surgery is the most frequently performed surgery in the world. Accurate measurement of ocular biometry and calculation of intraocular lens (IOL) implant power is crucial to achieving satisfactory postoperative refractive results. Important factors affecting the calculation of intraocular lens power are axial length (AL), anterior chamber depth (ACD) and keratometry (K). Gender differences in ocular biometry have been demonstrated by previous studies [1–5], with women on average having steeper corneas and shorter axial lengths than men. However, the finding that gender can play a role in biometry prediction error and intraocular lens power calculation is controversial [6–8]. While previous studies have indicated a relationship between gender and the prediction error (PE) of modern IOL formulas, questions remain.

In 2014, Behndig et al. demonstrated differences in the prediction errors between men and women for the SRK/T and Haigis formulas using data from the Swedish National Cataract Register (NCR). For SRK/T, a difference in both the sign and magnitude of the prediction errors for men and women were noted. Multiple factors warrant further evaluation, however, including: 1) the absence of optimized IOL constants in the NCR and 2) the absence of a commonly used fifth-generation formula such as Barrett Universal II for comparison.

This study was undertaken to provide more robust information about the effects of gender on ocular biometry and refractive prediction error. In undertaking this study, we also examine the potential effects of optimization of lens constants by gender to address disparities in refractive prediction errors with modern IOL formulas.

## Methods

### Data collection

Biometry records (including preoperative and postoperative biometry) between August 25, 2015 and June 27, 2019 were retrieved from Lenstar LS900 optical biometers (Haag-Streit USA Inc., EyeSuite software version i9.1.0.0) at University of Michigan’s Kellogg Eye Center. Institutional review board approval was obtained for the study and it was determined that informed consent was not required because of its retrospective nature and the anonymized data utilized in this study. Requirement of informed consent was waived by the University of Michigan Institutional Review Board. The study was carried out in accordance with the tenets of the Declaration of Helsinki. Patient demographics and cataract surgery information (including date of surgery and implanted IOL power) were obtained via the Sight Outcomes Research Collaborative (SOURCE) Ophthalmology Data Repository, which captures electronic health record (EHR) data of all patients receiving any eye care at academic medical centers participating in this research collaborative. SOURCE captures information on patient demographics, diagnoses identified based on International Classification of Diseases (ICD) codes, procedures based on Current Procedural Terminology (CPT) codes, and structured and unstructured (free-text) data from all clinical encounters (clinic visits, operative reports, etc.). For this study, we focused on a subset of the SOURCE patients receiving care at the University of Michigan.

Spherical equivalent manifest refractions from the postoperative month-one visit were identified from the clinical record for all patients who underwent cataract surgery (CPT = 66,984 or 66,982) from the dataset. Subjective non-mydriatic refractions were performed at the postoperative month-one visits among all patients who underwent cataract surgery in accordance with the standard protocol at Kellogg Eye Center. The power and model of the implanted intraocular lens for each surgery was collected as well. Only those surgeries involving the implantation of an Alcon SN60WF single-piece acrylic monofocal lens (Alcon, USA) were included in the study. Patients who had prior refractive surgeries were excluded from the dataset. Patients who had an additional surgery (e.g., endothelial keratoplasty) at the time of their cataract surgery were also excluded. Outliers in refractive prediction error, defined as greater than 1.96 standard deviations away from the mean, were excluded to address patients with unreliable refractions. Our final dataset included 8431 eyes of 5519 patients.

### Lens constant optimization

Lens constants were optimized for each formula to eliminate systematic errors in refraction prediction using previously described methods [9–11]. Patients were split in a 1:1 ratio into training and testing sets. The lens constants for five existing formulas (Barrett, Haigis, Hoffer Q, Holladay I, and SRK/T) were optimized utilizing the training set. Patients had either one or two records in the dataset depending on whether both eyes underwent surgery. One eye per patient was randomly kept in the testing dataset to ensure that each patient had the same weight during performance evaluation.

The refraction predictions of the Haigis, Hoffer Q, Holladay I, and SRK/T formulas were calculated according to their publications [12–19]. The prediction of the Barrett Universal II formula (hereafter referred to as Barrett) was obtained via the online calculator at https://calc.apacrs.org/barrett_universal2105/. The search space was centered around the default parameters given for the Alcon SN60WF by the Lenstar device. For all above formulas, the most optimal lens constant was defined as the one that minimized the absolute mean error in refraction prediction. In addition to optimization on the entirety of the training dataset, lens constant optimization was performed separately for women and men in the training set.

### Performance evaluation and statistical analysis

The refraction prediction errors of five modern IOL formulas (Barrett, Haigis, Hoffer Q, Holladay I, and SRK/T) were computed. Prediction error was calculated by subtracting postoperative refraction from the predicted refraction. Absolute predictive error was derived from the following formula: |predicted refraction - actual (postoperative) refraction|.

Statistical testing was performed to investigate relationships between variables in the dataset. A Wilcoxon rank sum test was performed using a single eye per patient to evaluate for differences in the means of biometry values and prediction errors between males and females. Presence of associations was tested with likelihood ratio tests from multivariate logistic regression. A Wilcoxon test was performed to assess the significance of the difference in the prediction errors when two different approaches were used for the lens constant optimization. Statistical significance was defined as *p*-value < 0.05. Statistical analyses were performed in R version 3.6.3 (R Core Team, Vienna, Austria).

## Results

### Demographics and dataset characteristics (Table 1)

Patient demographics and dataset characteristics are summarized in Table 1. Inclusion criteria were met by 8431 eyes, of which 4799 (56.9%) from women and 3632 (43.1%) from men. Women were found to be older on average at time of surgery than men by 0.51 years.

**Table 1:** Patient demographics and dataset characteristics

	Overall (***n*** = 8431)			Female (***n*** = 4799)			Male (***n*** = 3632)			***P***-value^**a**^
	Mean (SD)	Median	CI	Mean (SD)	Median	CI	Mean (SD)	Median	CI	
**Age at Surgery (Years)**	70.94 (9.72)	71.59	(70.73, 71.15)	71.16 (9.38)	71.77	(70.89, 71.42)	70.65 (10.15)	71.47	(70.32, 70.98)	0.028*
**Preoperative Measurements**										
AL (mm)	24.15 (1.37)	23.97	(24.12, 24.18)	23.90 (1.35)	23.71	(23.86, 23.94)	24.49 (1.32)	24.32	(24.44, 24.53)	< 0.0001*
CCT (μm)	551.36 (36.47)	551.00	(550.59, 552.14)	549.34 (36.29)	549.00	(548.31, 550.37)	554.04 (36.53)	553.00	(552.85, 555.23)	< 0.0001*
AD (mm)	2.69 (0.41)	2.70	(2.68, 2.70)	2.64 (0.40)	2.65	(2.63, 2.65)	2.76 (0.42)	2.78	(2.75, 2.77)	< 0.0001*
ACD (mm)	3.24 (0.41)	3.25	(3.24, 3.25)	3.19 (0.40)	3.20	(3.18, 3.20)	3.31 (0.42)	3.33	(3.30, 3.33)	< 0.0001*
LT (mm)	4.54 (0.45)	4.53	(4.53, 4.55)	4.53 (0.44)	4.52	(4.51, 4.54)	4.55 (0.47)	4.55	(4.54, 4.57)	0.235
Km (D)	43.86 (1.64)	43.84	(43.82, 43.89)	44.17 (1.58)	44.16	(44.12, 44.21)	43.44 (1.63)	43.38	(43.39, 43.49)	< 0.0001*
AST (D)	0.93 (0.81)	0.74	(0.92, 0.95)	0.91 (0.77)	0.73	(0.88, 0.93)	0.97 (0.86)	0.76	(0.94, 1.00)	0.039*
WTW (mm)	12.12 (0.53)	12.13	(12.11, 12.13)	12.04 (0.50)	12.05	(12.02, 12.05)	12.24 (0.54)	12.26	(12.22, 12.25)	< 0.0001*
**Postoperative Measurements**										
SPH (D)	− 0.87 (1.02)	− 0.66	(− 0.90, − 0.85)	− 0.91 (1.01)	− 0.66	(− 0.94, − 0.89)	− 0.82 (1.04)	− 0.66	(− 0.85, − 0.79)	< 0.0001*
CYL (D)	0.65 (0.69)	0.50	(0.64, 0.67)	0.63 (0.66)	0.50	(0.61, 0.64)	0.68 (0.72)	0.50	(0.66, 0.71)	0.006*
**Postoperative Refraction (D)**	−0.55 (0.96)	− 0.41	(− 0.57, − 0.53)	−0.60 (0.95)	− 0.41	(− 0.63, − 0.57)	−0.48 (0.97)	− 0.29	(− 0.51, − 0.45)	< 0.0001*

* Indicates statistical significance at the 0.05 level

^a^ Association between gender and continuous variables was assessed using the Wilcoxon rank sum test

### Preoperative measurements (Table 1)

Preoperative optical biometry parameters included axial length (AL), central corneal thickness (CCT), aqueous depth (AD), anterior chamber depth (ACD), lens thickness (LT), mean corneal curvature radius (Km), astigmatism (AST), and white-to-white corneal diameter (WTW). There was a statistical difference for every preoperative biometric measurement except lens thickness between females and males (Table 1).

The mean preoperative AL of patients was 24.15 ± 1.37 mm. The mean AL in males (24.49 ± 1.32 mm) was significantly longer than that in females (23.90 ± 1.35 mm). The mean ACD was 3.24 ± 0.41 mm. The mean ACD in males (3.31 ± 0.42 mm) was significantly deeper than in females (3.19 ± 0.40 mm). Overall, the preoperative AL, AD, CCT, ACD, AST and WTW in male patients were significantly larger than those in females (*p* < 0.05 for each). The mean Km of patients was 43.86 ± 1.64 D. Unlike other preoperative biometric measurements, the mean Km in females (44.17 ± 1.58 D) was significantly higher than males (43.44 ± 1.63 D).

### Postoperative measurements (Table 1)

Postoperative measurements included spherical (SPH) cylindrical (CYL) refraction components and spherical equivalent refraction. Mean postoperative CYL in males (0.68 ± 0.72 D) was higher than that of females (0.63 ± 0.66 D). Postoperative spherical equivalent refraction in females (− 0.60 ± 0.95 D) was more myopic than that of males (− 0.48 ± 0.97 D) irrespective of refractive target.

### Signed prediction error and absolute prediction error (Table 2)

Signed prediction error (SPE) was significantly different between male and female eyes (*p* < 0.0001), with errors skewed towards hyperopia for males and myopia for females for all five formulas considered. Absolute prediction errors (APE) were not significantly different between females and males for all five formulas.

**Table 2:** Gender differences in signed and absolute prediction errors

	Overall (*n* = 8431) Mean (SD)	Female (*n* = 4799) Mean (SD)	Male (*n* = 3632) Mean (SD)	*P*-value^a^
**Refractive Prediction Error**				
Holladay 1	− 0.017 (0.569)	0.053 (0.585)	− 0.110 (0.533)	< 0.0001*
SRK/T	−0.011 (0.570)	0.072 (0.585)	−0.120 (0.529)	< 0.0001*
Hoffer Q	−0.016 (0.596)	0.051 (0.623)	−0.104 (0.547)	< 0.0001*
Haigis	−0.019 (0.557)	0.011 (0.585)	−0.059 (0.514)	< 0.0001*
Barrett	−0.021 (0.516)	0.021 (0.533)	−0.076 (0.488)	< 0.0001*
**Refractive Absolute Prediction Error**				
Holladay 1	0.396 (0.409)	0.398 (0.432)	0.394 (0.375)	0.93
SRK/T	0.403 (0.403)	0.405 (0.428)	0.400 (0.367)	0.77
Hoffer Q	0.424 (0.420)	0.433 (0.451)	0.412 (0.375)	0.11
Haigis	0.386 (0.402)	0.391 (0.436)	0.379 (0.353)	0.30
Barrett	0.357 (0.374)	0.363 (0.391)	0.349 (0.349)	0.17

* Indicates statistical significance at the 0.05 level

^a^ Association between gender and continuous variables was assessed using Student t-test

### Regression analysis (Table 3)

Multivariate regression analysis was performed where the association of prediction error, as the dependent variables, was tested against each preoperative biometric measurement and gender as independent variables. Regression analysis was performed on the prediction error of five formulas, including Holladay 1, SRK/T, Hoffer Q, Haigis and Barrett. Only AL, Km, AST, age and gender had statistically significant association with prediction error in all five formulas.

**Table 3:** Regression Analysis for Variables Predicting Refractive Predictive Error

Formula	Significant Variables
Holladay	AL (−0.08), LT (−0.26), Km (−0.013), AST (− 0.065), WTW (− 0.081), Age (0.0037), Gender (− 0.054)
SRK/T	AL (0.013), LT (− 0.28), Km (0.097), AST (− 0.063), WTW (− 0.086), Age (0.0033), Gender (− 0.055)
Hoffer Q	AL (−0.14), LT (− 0.30), Km (− 0.11), AST (− 0.058), WTW (− 0.083), Age (0.0020), Gender (− 0.068)
Haigis	AL (−0.20), LT (− 0.32), Km (− 0.11), AST (− 0.031), WTW (− 0.084), Age (0.0017), Gender (− 0.077)
Barrett	AL (−0.030), LT (− 0.10), AST (− 0.069), Age (0.0023), Gender (− 0.057)

Coefficients in parentheses

### Lens constant optimization (Tables 4 and 5)

Lens constants optimization were performed with two approaches: 1) optimized based on all patients in the training set; and 2) optimized separately for male and female patients. Values of the optimized constants were shown in Table 4. When lens constants were optimized by gender, all five formulas demonstrated an improvement in prediction accuracy as measured by the mean absolute error (MAE) (Table 5). The overall reduction in MAE ranged between 0.16 and 2.25%. The improvements were statistically significant for SRK/T and Hoffer Q based on a Wilcoxon test (Table 5).

**Table 4:** Lens constants after standard optimization and optimization based on gender

		Standard Optimization	Optimization by Gender	
			Male	Female
Formula	Constant	A Constant	A Constant	A Constant
**Holladay1**	Surgeon Factor	1.867	1.952	1.805
**SRK/T**	A constant	119.093	119.264	118.977
**Hoffer Q**	ACD	5.722	5.802	5.665
**Haigis**	a0	−0.737	−0.694	−0.766
**Barrett**	Lens Factor	1.95	2.01	1.91

**Table 5:** Mean Absolute Error (MAE) after standard optimization and optimization by gender

Formula	Mean Absolute Error		% Reduction in Mean Absolute Error	*P*-value^a^
	Standard Optimization	Optimized by Gender		
Holladay	0.341	0.337	1.177	0.15
SRK/T	0.348	0.340	2.249	0.02*
Hoffer Q	0.367	0.363	1.074	0.002*
Haigis	0.335	0.334	0.162	0.46
Barrett	0.308	0.307	0.418	0.23

*Indicates statistical significance at the 0.05 level

^a^ Differences between Mean Absolute Error (MAE) after standard optimization versus optimization by gender was evaluated with Wilcoxon test

## Discussion

Gender differences in ocular biometry have long been studied in large populations and exist regardless of race and geographical location [2–5, 20] Our findings align with previous studies and indicate that women have smaller axial lengths [2–4, 21], anterior chamber depths, and horizontal white-to-white distances [2], in addition to greater mean keratometry than men (*p* < 0.0001) [2–4, 21]. Less studied ocular biometry measurements (including central corneal thickness and crystalline lens thickness) were higher in men (*p* < 0.05).

There is conflicting evidence in the literature, however, regarding the effect of gender on prediction error of IOL power calculation formulas. SRK/T was previously reported to generate a myopic prediction error for women, while subsequent studies reported no statistically significant gender differences in prediction error [7, 8, 21, 22].

In order to investigate this further, we gathered a large cataract surgery dataset (8431 eyes of 5519 patients) and performed lens-constant optimization using standard techniques over a training set consisting of 50% of the dataset. We found that for each of the 5 modern formulas considered (including Barrett Universal II), prediction error was significantly different between men and women (*p* < 0.0001 for each formula), with hyperopic errors for men and myopic errors for women. SRK/T demonstrated a prediction error of 0.072 ± 0.585 for women, and a prediction error of − 0.120 ± 0.529 for men despite standard lens constant optimization. Barrett also demonstrated significantly different prediction errors for women and men, skewed in different directions (prediction error of 0.021 ± 0.533 for women, and a prediction error of − 0.076 ± 0.488 for men) despite lens constant optimization.

The consistent finding of prediction errors with different signs between women and men across each of the 5 formulas raised the question of whether shorter eyes in women could explain the differences seen. In order to evaluate whether the differences in prediction error between men and women could be explained wholly by differences in ocular size, regression analyses of prediction error against ocular biometry were performed for each formula, including patient gender as a parameter. For each formula, however, patient gender was found to be an independent predictor of formula prediction error (*p* < 0.0001 for each formula).

Prior to this study, only preoperative AL, ACD, CCT and age had been shown to be independent predictors of differences in prediction error [23]. Our findings indicate that gender, age and preoperative AL, Km and AST were significant predictors of differences in prediction error among the 5 formulas studied. While both gender and age were found to be independent predictors of prediction error, the coefficients for gender were an order of magnitude higher than those of age at surgery, leading us to focus on the impact of gender on prediction error on this dataset.

The identification of gender as an independent predictor of formula prediction error, as well as the information regarding AL and Km encapsulated by gender, led to the consideration of optimizing lens constants separately based on gender.

To our knowledge, no prior studies have explored optimization of lens constants by gender despite clear differences in ocular biometry between men and women. In order to evaluate the potential impact of gender-based lens constants, optimization of lens constants was performed separately for women and men on the training set. The prediction errors of each formula were then computed on the testing set, using the lens constants for the general population and the gender-based lens constants. Optimization of lens constants by gender was found to significantly (*p* < 0.05) reduce mean absolute errors in refraction prediction for SRK/T and Hoffer Q formulas. Thus, our study demonstrates that optimization of lens constants by gender has the potential to improve refractive outcomes after cataract surgery when used with certain IOL power formulas.

The limitations of our study include the use of a retrospective, rather than prospective, dataset with little variation in race. All patients were extracted from a single institution in the United States. It was not possible compute predicted refraction, and thus refraction error, using the Hill-RBF or Holladay 2 methods, as mechanisms for bulk computation of these methods are not publicly available. In addition, it was not possible to exclude all potential ocular conditions that could affect the prediction errors of each formula. While multiple types of ocular pathology could affect predictions, we focused on excluding patients who had simultaneous surgeries and prior corneal refractive surgeries that altered the corneal curvature.

The finding that gender was an independent predictor of refraction prediction error for all 5 formulas studied indicates that incorporating gender into these (and potentially other) methods of IOL selection can decrease prediction error overall. While we studied the effects of incorporating gender into the lens constant, the development of additional methods for reducing disparities in prediction error warrants further attention.

While alternative methods of incorporating gender into IOL power calculation formulas may yield greater reductions in absolute prediction error, the simplicity of implementing gender-based lens constants makes it a potential option for improving cataract surgery refractive outcomes. No additional measurements or devices are needed. On the Lenstar device, which was utilized for this study, separate calculation templates could be created for each gender utilizing customized lens constants, thus requiring only one additional click to achieve a 0.16–2.25% reduction in refractive error after cataract surgery using existing IOL calculation methods.

## Conclusions

Gender was found to be an independent predictor of refraction prediction error for all 5 modern IOL formulas studied. Implementing lens constants optimized by gender can decrease refraction prediction errors and may reduce disparities in cataract surgery outcomes.

## Data Availability

The datasets generated and/or analyzed during the current study are not publicly available to protect patient privacy but are available from the corresponding author on reasonable request.

## Abbreviations

ACD: Anterior chamber depth
AD: Aqueous depth
AL: Axial length
APE: Absolute prediction error
AST: Astigmatism
CCT: Central corneal thickness
CPT: Current Procedural Terminology
EHR: Electronic health record
ICD: International Classification of Diseases
IOL: Intraocular lens
K: Keratopathy
Km: Mean corneal curvature radius
LT: Lens thickness
NCR: Swedish National Cataract Register
PE: Prediction error
SOURCE: Sight Outcomes Research Collaborative
SPE: Signed prediction error
WTW: White-to-white corneal diameter
